# Intermittency and Self-Organisation in Turbulence and Statistical Mechanics

**DOI:** 10.3390/e21060574

**Published:** 2019-06-06

**Authors:** Eun-jin Kim

**Affiliations:** School of Mathematics and Statistics, University of Sheffield, Sheffield S3 7RH, UK; e.kim@sheffield.ac.uk

**Keywords:** turbulence, statistical mechanics, intermittency, coherent structure, multi-scale problem, self-organisation, bifurcation, non-locality, scaling, multifractal

There is overwhelming evidence, from laboratory experiments, observations, and computational studies, that coherent structures can cause intermittent transport, dramatically enhancing transport. A proper description of this intermittent phenomenon, however, is extremely difficult, requiring a new non-perturbative theory, such as statistical description. Furthermore, multi-scale interactions are responsible for inevitably complex dynamics in strongly non-equilibrium systems, a proper understanding of which remains one of the main challenges in classical physics. However, as a remarkable consequence of multi-scale interaction, a quasi-equilibrium state (so-called self-organisation) can be maintained.

This Special Issue presents different theories of statistical mechanics to understand this challenging multiscale problem in turbulence. The 14 contributions to this Special Issue focus on the various aspects of intermittency, coherent structures, self-organisation, bifurcation and nonlocality. Given the ubiquity of turbulence, the contributions cover a broad range of systems covering laboratory fluids (channel flow, the Von Kármán flow), plasmas (magnetic fusion), laser cavity, wind turbine, air flow around a high-speed train, solar wind and industrial application. The following is a short summary of each contribution.

Mathur et al. [1] address the importance of structures in the transient behaviour of a channel flow at high Reynolds number Re. Large-eddy simulations of turbulent channel flow subjected to a step-like acceleration reveal the transition of transient channel flow comprised of a three-stage response similar to that of the bypass transition of boundary layer flows; the effect of the structures (the elongated streaks) becomes more important in the transition for large Re. Their analysis employing conditionally-averaged turbulent statistics elucidates the interplay between structures and active/inactive regions of turbulence depending on Re.

Chliamovitch and Thorimbert [2] present a new method of dealing with non-locality of turbulence flows through the formulation of the bilocal kinetic equation for pairs of particles. Based on a maximum-entropy-based generalisation of Boltzmann’s assumption of molecular chaos, they utilise the two-particle kinetic equations and derive the balance equations from the bilocal invariants to close their kinetic equations. The end product of their calculation is non-viscous hydrodynamics, providing a new dynamical equation for the product of fluid velocities at different points in space.

Jacquet et al. [3] address the formation of coherent structures and their self-organisation in a reduced model of turbulence. They present the transient behaviour of self-organised shear flows by solving the Fokker–Planck equation for time-dependent Probability Density Functions (PDFs) and model the formation of self-organisation shear flows by the emergence of a bimodal PDF with the two peaks for non-zero mean values of a shear flow. They show that the information length—The total number of statistically different states that a system passes through in time—is a useful statistical measure in understanding attractor structures and the time-evolution out of equilibrium.

Xu et al. [4] deal with an unsteady flow in wind turbines and show the importance of structures (turbulent winds/wind shears) on the stability of the floating wind. Based on the vortex theory for the wake flow field of the wind turbine, they invoke the Free Vortex Wave method to calculate the rotor power of the wind turbine. Depending on the turbulent wind, wind shear, and the motions of the floating platform, they put forward a trailing-edge flap control strategy to reduce rotor power fluctuations of a large-scale offshore floating wind turbine. Their proposed strategy is shown to improve the stability of the output rotor power of the floating wind turbine under the turbulent wind condition. 

Anderson et al. [5] model anomalous diffusion and non-local transport in magnetically confined plasmas by using a non-linear Fractional Fokker–Planck (FFP) equation with a fractional velocity derivative. Their model is based on the Langevin equation with a nonlinear cubic damping and an external additive forcing given by a Lévy-stable distribution with the fractality index α (0 < α < 2). By varying α, they numerically solve the stationary FFP equation and analyse the statistical properties of stationary distributions by using the Boltzmann–Gibbs entropy, Tsallis’ q-entropy, q-energies, and generalised diffusion coefficient, and show the significant increase in transport for smaller α.

Saini et al. [6] highlight key challenges in modelling high Reynolds number unsteady turbulent flows due to complex multi-scale interactions and structures (e.g., near wall) and discuss different advanced modelling techniques. Given the limitation of the traditional Reynolds-Averaged Navier–Stokes (RANS) based on stationary turbulent flows, they access the validity of the Improved Delayed Detached Eddy Simulation (IDDES) methodology using two different unsteady RANS models. By investigating different types of flows including channel (fully attached) flow and periodic hill (separated) flow at different Reynolds numbers, they point out the shortcomings of the IDDES methodology and call for future work.

Barbay et al. [7] address the formation of oscillatory patterns (structures), bifurcations and extreme events in an extended semiconductor microcavity laser. Experimentally, as an example of self-pulsing spatially extended systems, they consider vertical-cavity surface emitting lasers with an integrated saturable absorber and study the complex dynamics and extreme events accompanied by spatiotemporal chaos. Theoretically, by employing the Ginzburg–Landau model, they characterize intermittency by the Lyapunov spectrum and Kaplan–Yorke dimension and show the chaotic alternation of phase and amplitude turbulence, extreme events induced by the alternation of defects and phase turbulence.

Wang et al. [8] investigate the effect of streaks (structures) on wall-bounded turbulence at low-to-moderate Reynolds number by using 2D Particle Image Velocimetry measurement and direct numerical simulations. To understand the spanwise spacing of neighbouring streaks, they present a morphological streak identification analysis and discuss wall-normal variation of the streak spacing distributions, fitting by log-normal distributions, and Re-(in)dependence. They then reproduce part of the spanwise spectra by a synthetic simulation by focusing on the Re-independent spanwise distribution of streaks. Their results show the important role of streaks (structures) in determining small-scale velocity spectra beyond the buffer layer. 

Van Milligen et al. [9] address the importance of self-organisation and structures in transport in magnetically confined fusion plasmas far from equilibrium by studying the radial heat transport in strongly heated plasmas. By using the transfer entropy, they identify the formation of weak transport barriers near rational magnetic surfaces most likely due to zonal flows (structures) and show that jumping over transport barriers is facilitated with the increasing heating power. The behaviour of three different magnetic confinement devices is shown to be similar. They invoked a resistive magneto-hydrodynamic (fluid) model and continuous-time random walk to understand the experiment results.

He et al. [10] address turbulence in the air over a high-speed train and the formation of a coherent structure near the vent of a train, which plays an important role in the dissipated energy through the skin friction. By modelling the ventilation system of a high-speed train by a T-junction duct with vertical blades, they calculate the velocity signal of the cross-duct in three different sections (upstream, mid-center and downstream), and analyse the coherent structure of the denoised signals by using the continuous wavelet transform. Results show that the skin friction of the train decreases with the increasing ratio of the suction velocity of ventilation to the velocity of the train. 

Alberti et al. [11] discuss turbulence, intermittency and structure in the solar wind by using fluid (magnetohydrodynamic) and kinetic approaches. By analysing solar wind magnetic field measurements from the ESA Cluster mission and by using the empirical mode decomposition based multi-fractal analysis and a chaotic approach, they investigate self-similarity properties of solar wind magnetic field fluctuations at different timescales and the scaling relation of structure functions at different orders. The main results include multi-fractal and mono-fractal scalings in the inertial range and the kinetic/dissipative range, respectively. 

Geneste et al. [12] address intermittency in high Reynolds number turbulence by studying the universality of the multi-fractal scaling of structure function of the Eulerian velocity. Experimentally, they measure the radial, axial and azimuthal velocity in a Von Kármán flow, using the Stereoscopic Particle Image Velocimetry technique at different resolutions while performing direct numerical simulations of the Navier-Stokes equations. They demonstrate a beautiful log-universality in structure functions, link it to multi-fractal free energy based on the analogy between multi-fractal and classical thermodynamics and invoke a new idea of a phase transition related to fluctuating dissipative time scale.

Podgórska [13] discuss the effect of internal (fine-scale) intermittency due to vortex stretching on liquid–liquid dispersions in a turbulent flow with applications to industry. The internal intermittency is related to a strong local and instantaneous variability of the energy dissipation rate, and the k-ε model and multifractal formalism are used to understand turbulence properties and internal intermittency in droplet breakage and coalescence. By solving the population balance equation and CFD simulations, they elucidate the effects of the impeller type—six-blade Rushton turbine and three-blade high-efficiency impeller—and droplet breakage coalescence (dispersion) on drop size distribution.

De Divitiis [14] review their previous works on homogenous isotropic turbulence for incompressible fluids and a specific (non-diffusive) Lyapunov theory for closing the von Kármán–Howarth and Corrsin equations without invoking the eddy-viscosity concepts. In particular, they show that the bifurcation rate of the velocity gradient along fluid particle trajectories exceeds the largest Lyapunov exponent and that the statistics of finite-time Lyapunov exponent of the velocity gradient follows normal distributions. They also discuss the statistics of velocity and temperature difference by utilising a statistical decomposition based on extended distribution functions and the Navier–Stokes equations.
